# Lifestyle counseling in hypertension-related visits – analysis of video-taped general practice visits

**DOI:** 10.1186/1471-2296-9-58

**Published:** 2008-10-14

**Authors:** Ivon EJ Milder, Anneke Blokstra, Judith de Groot, Sandra van Dulmen, Wanda JE Bemelmans

**Affiliations:** 1 RIVM (National Institute of Public Health and the Environment), Centre for Prevention and Health Services Research, Bilthoven, The Netherlands; 2 NIVEL (Netherlands Institute for Health Services Research), Utrecht, The Netherlands

## Abstract

**Background:**

The general practitioner (GP) can play an important role in promoting a healthy lifestyle, which is especially relevant in people with an elevated risk of cardiovascular diseases due to hypertension. Therefore, the aim of this study was to determine the frequency and content of lifestyle counseling about weight loss, nutrition, physical activity, and smoking by GPs in hypertension-related visits. A distinction was made between the assessment of lifestyle (gathering information or measuring weight or waist circumference) and giving lifestyle advice (giving a specific advice to change the patient's behavior or referring the patient to other sources of information or other health professionals).

**Methods:**

For this study, we observed 212 video recordings of hypertension-related visits collected within the Second Dutch National Survey of General Practice in 2000/2001.

**Results:**

The mean duration of visits was 9.8 minutes (range 2.5 to 30 minutes). In 40% of the visits lifestyle was discussed (n = 84), but in 81% of these visits this discussion lasted shorter than a quarter of the visit. An assessment of lifestyle was made in 77 visits (36%), most commonly regarding body weight and nutrition. In most cases the patient initiated the discussion about nutrition and physical activity, whereas the assessment of weight and smoking status was mostly initiated by the GP. In 35 visits (17%) the GP gave lifestyle advice, but in only one fifth of these visits the patient's motivation or perceived barriers for changing behavior were assessed. Supporting factors were not discussed at all.

**Conclusion:**

In 40% of the hypertension-related visits lifestyle topics were discussed. However, both the frequency and quality of lifestyle advice can be improved.

## Background

The prevalence of hypertension is high and in the Netherlands it is the most common reason to visit the general practitioner [1]. Healthy lifestyle is important in the prevention of cardiovascular diseases, especially for people with elevated risk due to hypertension. The general practitioner (GP) can play an important role in health promotion. Several studies have shown that lifestyle advice given by the GP can be effective in changing lifestyle [2-5]. However, well known barriers for performing behavioral counseling are poor compliance by patients, lack of time, and insufficient knowledge about the topics [6-8] Especially with regard to losing weight and increasing physical activity evidence exists that the quality of counseling is not optimal, and opportunities are missed [9-12]. Some of the established quality criteria for lifestyle counseling are that it consist of goal setting, is individually targeted, and includes an assessment of the patients motivation and potential barriers and supporting factors [13,14]. However, data about the incorporation of these counseling elements in daily family practice are not available.

According to national [15] and international [16] guidelines, lifestyle should be included in the assessment and management of cardiovascular disease risk of hypertension patients. In a previous study which investigated adherence to the national hypertension guideline, 80% of the Dutch GPs reported that they implemented the guideline, including an assessment of smoking status, body mass index (BMI), and physical activity [17]. In addition, they reported that they always or often gave lifestyle advice on these topics. Two other studies confirmed a high self-reported adherence to guidelines, and the lifestyle counseling as part of them [18,19]. However, these previous studies are based on self-report by the GPs in questionnaires, and thus GPs may overestimate the frequency of lifestyle counseling. Furthermore, these studies do not provide insight in the actual content and quality of the counseling.

Therefore, in the present study, we have observed video-recordings of 212 hypertension-related visits, recorded in 2000/2001 as part of the Second National Survey of General Practice [1,20]. This makes it possible to observe what GPs and patients actually discuss in practice. The aim of this study was to determine the frequency, content and quality of lifestyle counseling (about weight, nutrition, physical activity, and smoking) in hypertension-related visits.

## Methods

### Population

This study is based on a standardized observation of lifestyle discussions during GP visits which were video recorded as part of the Second Dutch National Survey of General Practice. This is a large study, which investigates several aspects of primary care [1,20,21] The main aim of the video registrations was to determine the GPs' style of communication. Neither GPs nor patients were aware that lifestyle counseling was a topic of interest for the researchers.

The larger study group consisted of a representative sample of 195 Dutch GPs of which 142 agreed to have 15–20 of their visits recorded on videotape. Per GP these visits were recorded with an unmanned camera over one or two successive days in 2000 or 2001. The study was carried out according to Dutch privacy legislation. The privacy regulation was approved by the Dutch Data Protection Authority. According to Dutch legislation, approval by a medical ethics committee was not required for this observational study. All participating GPs and patients have granted written consent prior to the visit. In addition, all patients had the possibility to withdraw consent after the visits, which would lead to a deletion of all their data. None of the patients withdrew consent after the visit. Due to the position of the camera the patients were only visible at the back of the head or not visible at all. Eighty-eight percent of the patients agreed to participate. In total 2784 visits were recorded [22]. We have included all hypertension-related visits.

Previously, the four major complaints of the patients were coded by the observers according to the International Classification of Primary Care (ICPC) [1]. For the present study we have selected visits of patients aged 18 years or older with ICPC codes K85 'High blood pressure' and K86 'Essential hypertension without organ damage' as first, second, or third complaint. There were 145 visits with ICPC K85 or K86 as first complaint, 82 as second complaint, and 18 as third complaint. This resulted in 245 visits.

According to the Dutch hypertension guideline the blood pressure should be measured eight to twelve times during four to six visits. If the mean of the blood pressure measurements (excluding those made during the first visit) is >140 mg Hg for systolic and/or >90 mm Hg diastolic the diagnosis hypertension is made [15]. In the present study, we included patients with established hypertension, as well as patients with elevated blood pressure in one or more visits for whom the diagnosis hypertension was not (yet) made. Furthermore, we included patients who had a blood pressure measurement because of other reasons, such as a general medical check-up or diabetes, when we could derive from the video that their blood pressure was elevated (systolic blood pressure ≥ 140 mm Hg or diastolic ≥ 90 mm Hg or the GP mentioned that the blood pressure was too high) (n = 27). We have excluded 16 visits, in which patients neither had established hypertension, nor elevated blood pressure, and 17 visits in which we could not establish the patient's blood pressure status because their blood pressure was not mentioned. So, in total 212 of the 245 observed visits were included, one to seven visits per GP. (mean ± SD: 2.0 ± 1.3).

### Data collection

Preceding the visit, and two weeks after the visit, patients completed questionnaires on sociodemographic characteristics and health status. Observation of the videotapes was done by two observers using a for this specific purpose developed protocol. The protocol was specifically set up to evaluate the compliance to the lifestyle component of the Dutch GP hypertension guideline [15], and was further based on theories of changing behavior, in particular the theory of planned behavior [13].

The protocol included general information such as total duration of the visit, main topics (according to the duration of the discussion), and the duration of the discussion of lifestyle. The lifestyle topics included were weight management, nutrition, physical activity, and smoking. We coded whether the GP or the patient initiated the discussion about lifestyle. The content of lifestyle counseling was classified as assessment of lifestyle or giving specific advice.

*Assessment of lifestyle *was defined as information gathering on weight, or weight change, dietary pattern, physical activity, or smoking status, or (recent) changes in these behaviors. It also included the measurements of body weight or waist circumference. Discussion of alcohol use was regarded as a discussion of 'nutrition', unless the focus was on problems related to alcohol addiction.

*Giving lifestyle advice *was defined as giving a specific advice or information on these topics, referring the patient to other information sources, handing out written materials, or referring the patient to a dietitian, practice assistant or stop-smoking course.

Furthermore, the 'quality' of lifestyle counseling was assessed by observing whether certain elements of lifestyle counseling were used by the GP. The elements included in the protocol were: assessment of the patient's motivation for the behavior change; assessment of the patient's confidence to perform the behavior change; assessment of perceived barriers or supporting factors; setting of specific behavior change goals; and planning a follow-up appointment for the evaluation of the behavior change.

Difficulties in coding were resolved by discussion between the observers, using their notes made during the observation. To determine the interrater agreement, a random sample of eleven visits was coded by both observers. Pearson correlation coefficients between the two coders were determined for all categories that were perceived at least twice. For the data reported in this paper (tables and figures), the Pearson correlation coefficients between the two coders ranged from 0.67 to 1.00.

### Statistics

Statistical analyses were performed using SPSS, release 12.0, Chicago, IL.

We determined the frequency of different elements of lifestyle counseling according to the above-mentioned criteria. Since the frequency of lifestyle counseling might differ between patients in a different stage of the diagnosis (established hypertension/early phase of diagnosis), age (<65y or >65y), and gender (male/female), we have determined the frequency of lifestyle counseling after stratification for these patient groups. In addition, we have evaluated whether the frequency of lifestyle counseling differed between male or female GPs. Differences between groups were tested using chi square tests.

## Results

### General characteristics of GPs and patients

The majority of the patients was over 65 years of age, and the patient population included more women than men (table 1). Most of the visits were regular check-ups of patients with established hypertension. The majority of the GPs was male. The most important topics during the visit (besides blood pressure) were medication; complaints of the musculoskeletal system; (results of) medical examination; family or social environment; psychological problems; skin, hair and nails; and the cardiovascular system

**Table 1:** Characteristics of the participants and general practitioners

	Percentage
**Patients (n = 212)**	
**Age**	
18–44 years	10
45–64 years	33
65–74 years	32
≥ 75 years	24
	
**Gender**	
Female	67
Male	33
	
**Nationality**	
Dutch	78
Other	13
Unknown	9
	
**Education**	
Non/elementary school	27
High school or vocational education	46
College or university	13
Unknown	14
	
**Blood pressure status**	
Established hypertension	78
Early phase†	9
Other‡	13
	
**GPs (n = 108)**	
**Age**	
<35 years	8
35–44 years	32
45–54 years	52
>55 years	7
	
**Gender**	
Female	23
Male	77
	
**Urbanization practice location**	
Moderately/strongly	59
Weakly/not	41
	
**Employment**	
≥ 0.8 full-time equivalents	72
< 0.8 full-time equivalents	28
	
**Practice**	
Single	33
Duo	29
Group	38

† Elevated blood pressure in one or more visits but diagnosis hypertension not (yet) established.

‡ E.g. general medical check-up, diabetes.

### Frequency and duration of lifestyle counseling

**Figure 1:**
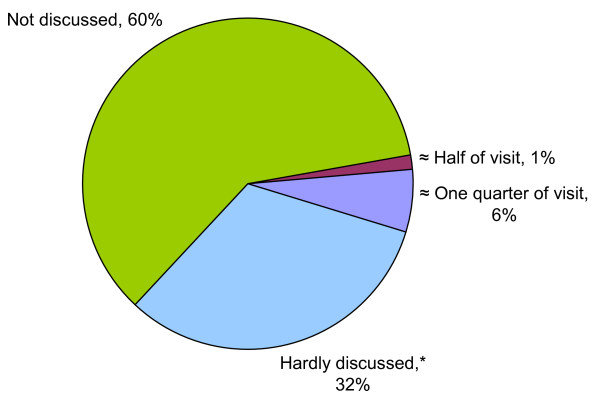
**Duration of lifestyle counseling**. * Less than a quarter of the visit.

The mean ± SD duration of complete visits was 9.8 ± 4.7 minutes, ranging from 2.5 to 30 minutes. A lifestyle topic was discussed in 40% of the visits. In most cases, the duration of lifestyle counseling was brief (figure 1). In 7% of the visits lifestyle counseling lasted a quarter of the visit or more, and in 1% this was half the visit or more. The duration of the visit tended to be longer when lifestyle was discussed than when it was not discussed (mean duration ± SD: 10.4 ± 4.3 minutes vs 9.5 ± 4.8 minutes; *P *= 0.57).

### Assessment of lifestyle

**Figure 2:**
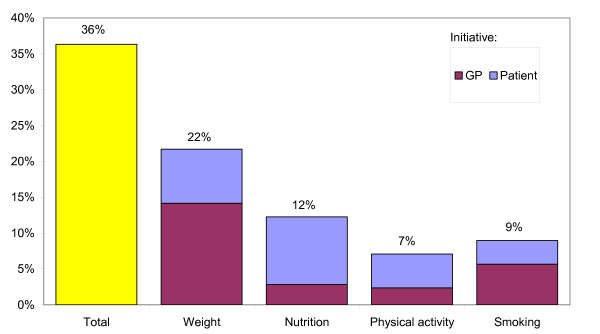
Percentage of visits in which an assessment of lifestyle was performed.

In 36% of the visits an assessment of lifestyle was performed (figure 2). Weight was assessed most often (22% of the visits), followed by nutrition (12%). In most cases the GP initiated the discussion about weight and smoking, whereas for nutrition and physical activity it was mostly initiated by the patient.

Most of the visits in which the patients' lifestyle was assessed, regarded only one of the four lifestyle topics. In 16 visits two of the lifestyle topics were assessed. The most common combination was an assessment of weight and nutrition (9 visits). In two visits three of the lifestyle components were assessed. One of these visits included an assessment of weight, nutrition, and smoking, the other weight, physical activity and smoking.

### Specific lifestyle advice

**Figure 3:**
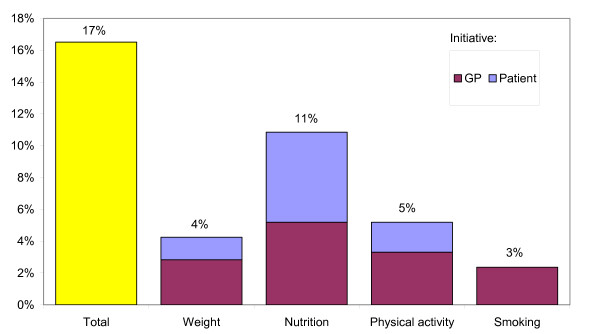
Percentage of visits in which the GP gave a specific lifestyle advice to change behavior.

In 35 of the 212 visits (17%) the GP gave advice or information on weight loss, nutrition, physical activity, or smoking (figure 3). Besides the advice given by the GP, two patients received written information and four patients were referred to a practice assistant. Most of the advice regarded nutrition; this included three cases in which the patient was referred to a dietitian. Advice on nutrition was mostly given after the patient initiated the discussion on this topic, whereas the discussions about weight, physical activity, and smoking were mostly initiated by the GP.

In 25 of the 35 visits in which lifestyle advice was given only one of the four lifestyle topics was discussed. In 7 visits advice on two topics was given; three times weight loss and nutrition were combined, three times nutrition and physical activity; and one time physical activity and smoking. In three visits advice on three topics was combined (weight loss, nutrition and physical activity).

In most cases, the content of lifestyle advice was quite general. A few examples of the advice given are 'Try to loose some weight, you are too heavy', 'Limit your use of salt', 'Limit your alcohol use to a maximum of two glasses [of whiskey] per day', 'Keep in shape, keep cycling', 'Smoking is an important risk factor, it is more beneficial to stop smoking than to take an extra anti-hypertension pill'.

We also observed how often an assessment was combined with an advice during the same visit. In 9 of the 46 visits in which weight was assessed, also advice on weight loss was given; and in 15 of these visits advice on weight loss, nutrition and/or physical activity was given. The assessment of nutrition was followed by nutrition advice in 11 out of 26 visits. For physical activity and smoking these numbers were 3 out of 15 and 6 out of 19, respectively.

When advice on weight or smoking was given it was (almost) always preceded by an assessment of these factors. However, advice on nutrition or physical activity was most often given without an assessment of this factor (52% of the nutrition advice and 73% of the PA advice).

The patient's motivation for change was evaluated in only 6 of the 35 visits in which the GP gave lifestyle advice, and in 7 visits potential barriers for behavior change were discussed. In two of these visits the advice regarded smoking. In these visits the reported barriers were 'side effects of stopping and lack of confidence', and weight gain (after a previous cessation attempt), respectively. In one visit the advice regarded nutrition, and the reported barrier was 'not being able to eat less'. Four advices regarded physical activity. In all of these visits the reported barrier was physical inability to (increase) physical activity; three times due to physical limitations and once due to age of the patient. Social support and other supporting factors were not discussed at all, and follow-up appointments to discuss the lifestyle change(s) were never made.

In patients with established hypertension GPs less frequently made an assessment of lifestyle, and/or gave lifestyle advice than in patients in which the diagnosis hypertension was not (yet) made (table 2). There were no differences in the frequency of lifestyle counseling between patients younger or older then 65 years. Although there was no difference in the frequency of assessing lifestyle in male and female patients, male patients more often received advice than female patients. The frequency of all four types of advice was higher in men than in women, but only for advice on weight loss the difference was statistically significant (frequency 10% vs 1%, *P *= 0.004). There were no differences in the frequency of lifestyle counseling between male and female GPs. In addition, there were no differences in the frequency of lifestyle counseling between GPs for whom we observed one or two, or three and more visits.

**Table 2:** Percentage of visits which included lifestyle counseling, after stratification for stage of diagnosis, age, patient gender and GP gender

	Assessment	Advice	Total
**Stage of diagnosis**			
Established hypertension (n = 165)	32	12	35
Early phase† (n = 20)	45	25	50
Other/unknown (n = 27)	56	37	63
*P *(difference)	0.04	0.003	0.01
			
**Age**			
<65y (n = 92)	41	19	44
=>65y (n = 120)	33	15	37
*P *(difference)	0.19	0.50	0.32
			
**Patient gender**			
Male (n = 71)	39	24	42
Female (n = 141)	35	13	38
*P *(difference)	0.50	0.04	0.58
			
**GP gender (per visit)**			
Male (n = 157)	38	17	41
Female (n = 55)	33	14	36
*P *(difference)	0.52	0.65	0.57

† Elevated blood pressure in one or more visits but diagnosis hypertension not (yet) established.

## Discussion

The present study showed that, in hypertension-related GP visits, lifestyle counseling was performed in 40% of the observed visits. An assessment of lifestyle was performed in one third of the visits, most commonly for weight and nutrition, and less frequently for smoking and physical activity. In one sixth of the visits a recommendation to change behavior was made. Most of the advice regarded nutrition. The discussion of nutrition was most often initiated by the patient whereas the discussion of weight and smoking was most often initiated by the GP.

In most visits the duration of lifestyle counseling was brief, in only 7% of the visits it took more than a quarter of the visits. In addition, observation of the videotaped visits revealed that specific elements of lifestyle counseling were rarely or never used by the GPs. For example, the patient's motivation for a change in behavior and potential barriers or supporting factors were almost never discussed, and in none of the visits a follow-up visit for the evaluation of the behavior change was planned. This illustrates that the lifestyle advice was almost never specified and tailored to the specific situation of the patient. Thus, in most visits the discussion of lifestyle can actually not be characterized as lifestyle counseling in the strictest sense, but rather as a brief lifestyle advice (and/or assessment).

The frequency of lifestyle counseling in this study was lower than that reported in previous studies [17-19] However, in these studies the frequency of lifestyle counseling was determined using GP questionnaires, and thus GPs may have overestimated the frequency of lifestyle advice. In addition, in some of these studies response rates were low [17,18] which may have lead to selection bias. Another reason that the frequency of lifestyle counseling in our study is lower than that in previous studies may be that we have included only one visit per patient. According to the guidelines patients with hypertension have to visit their GP at least two times per year [15]. In previous studies it was assessed whether the GP gave lifestyle advice during the first hypertension-related visit [18,19] or at any given time [17].

Using video recordings of real life, everyday visits, we were able to determine what GPs actually do in practice. In this study the response rate was relatively high, 73% of the GPs and 88% of the patients agreed to have the visit recorded [20]. The study was primarily aimed at the communication between GPs and patients. So, both patients and GPs were not aware that lifestyle counseling would be one of the topics of investigation. Therefore it is not likely that GPs and/or patients have changed the frequency of lifestyle counseling because the visit was recorded on video.

A drawback of this study is that the current lifestyle of the patients was not known. For instance, it was not known whether the patient was a smoker or was overweight, unless this was mentioned during the visit. The GP can make an assessment of the patient's weight by looking at the patients, and may have knowledge about the lifestyle of the patient from previous visits. In addition, in the Second Dutch Survey of General Practice, the majority of the practice assistants indicated that they (sometimes) perform blood pressure measurements [23]. This may also be an opportunity for lifestyle counseling, but these patient contacts have not been included in the video recordings.

It is likely that the results may cluster per GP, and thus this should preferably be taken into account during the analyses, i.e. to perform multi-level analyses. However, for most (73%) of the GPs we included only one or two visits. Therefore, it was not feasible to perform multilevel analysis.

Our study was primarily aimed at the frequency of lifestyle counselling and the way it was delivered in practice. We did not focus on congruence of the actual contents of the assessment and advice with existing guidelines and/or other available evidence, but as mentioned previously most advices were brief and quite general. Due to the study design we could not evaluate whether certain counselling elements (e.g. assessment of the patient's motivation of the change in behaviour, goal-setting etc.) indeed increased the efficacy of the advice. Thus, further research is needed to evaluate the actual content and efficacy of the advice.

In 2001, approximately half of the GP patients was overweight (BMI > 25) [24]. Given the fact that overweight is one of the risk factors contributing to hypertension [25], we expect the prevalence of overweight in this group of patients to be even higher. According to the guidelines, if applicable, attention should be paid to the modifiable risk factors (smoking, weight and alcohol use) during every visit. However, in only one fifth of the visits an assessment of weight was made, and only nine patients received advice regarding their weight. This indicates that only a small proportion of the eligible patients have received counseling regarding their weight, and that the frequency of lifestyle counseling could be increased. To achieve this, barriers to lifestyle counseling, such as lack of time should be addressed. Since lack of time is one a the main barriers reported by GPs [6-8]., a potential solution may be that lifestyle counseling is (partly) performed by other health professionals, such as practice assistants or nurse physicians.

In this study, men more often received lifestyle advice than women, and this could be mainly contributed to a higher frequency of weight loss advice. This is partly contradicts results of previous research which showed that although men more often received lifestyle advice than women, [26,27], they were less often encouraged to lose weight [10,27,28].

A possible explanation for the higher frequency of lifestyle advice in men may be that GPs particularly target high-risk patients and men may be perceived to be at higher (cardiovascular) risk [26,27] On the other hand, it has been suggested that women are more likely to ask about lifestyle, particularly diet and weight, which may explain the contradictory results for weight loss advice [27]. In our study weight loss advice was mainly initiated by the GP, and thus it may also be explained by the GPs high-risk approach. As we have mentioned before, we did not have information on patient's weight status. Thus, the higher frequency of weight loss counseling in males may be explained by a higher prevalence of overweight in male than in female patients. However, this is not in line with our expectations, because in 2001 the prevalence of overweight in male primary care patients aged 55–74y was slightly lower than that in female patients [24].

The assessment of weight and smoking was more often initiated by the GP than the discussion of nutrition and physical activity. This can probably be explained by the fact that weight status and smoking are relatively easy to assess. Thus, probably nutrition and physical activity would be more frequently assessed if tools would become available for routine recording in a standardized way.

In 2006 the guidelines for Hypertension [15] and Cholesterol [29] were replaced by the guideline Cardiovascular risk management [30]. The recommended lifestyle advice in this standard are almost identical to those in the hypertension guideline. So we don't expect that the quality and frequency of lifestyle counseling of GPs was changed due to the revision of the guideline(s). However, a significant change compared to the previous (hypertension) guideline is that it is advised to refer motivated patients to a practice assistant, nurse practitioner, physiotherapist, dietician, behavioral counselor, a (stop smoking) course, or self-help program. In addition, it is likely that the attention for lifestyle of both GPs and patients has increased since 2001, because there seems to be a growing interest for this topic in general. The Third National Survey of General Practice that is currently in preparation provides an opportunity to study whether the frequency and quality of lifestyle counseling have changed in recent years.

## Conclusion

In less than half of the hypertension-related visits lifestyle topics were discussed. However, the duration of mostly brief, and it was not performed according to important principles of lifestyle counseling. Thus, both the frequency and quality of lifestyle counseling can be improved to increase efficacy.

## Competing interests

The authors declare that they have no competing interests.

## Authors' contributions

IEJM contributed to data analysis, and interpretation and writing the paper. AB contributed to data collection, analysis, and interpretation and writing the paper. JdG was responsible for data collection, interpretation, and commenting on drafts of the paper. SvD was responsible for designing the study, interpretation of the findings, and commenting on drafts of the paper. WJEB was responsible for design of the study and observation protocol, interpretation of the findings, and commenting on drafts of this paper. All authors have read and approved the final manuscript.

## Pre-publication history

The pre-publication history for this paper can be accessed here:


